# Adaptation and Selective Information Transmission in the Cricket Auditory Neuron AN2

**DOI:** 10.1371/journal.pcbi.1000182

**Published:** 2008-09-26

**Authors:** Klaus Wimmer, K. Jannis Hildebrandt, R. Matthias Hennig, Klaus Obermayer

**Affiliations:** 1 School of Computer Science and Electrical Engineering, Technische Universität Berlin, Berlin, Germany; 2 Bernstein Center for Computational Neuroscience, Berlin, Germany; 3 Department of Biology, Humboldt Universität zu Berlin, Berlin, Germany; Gatsby Computational Neuroscience Unit, United Kingdom

## Abstract

Sensory systems adapt their neural code to changes in the sensory environment, often on multiple time scales. Here, we report a new form of adaptation in a first-order auditory interneuron (AN2) of crickets. We characterize the response of the AN2 neuron to amplitude-modulated sound stimuli and find that adaptation shifts the stimulus–response curves toward higher stimulus intensities, with a time constant of 1.5 s for adaptation and recovery. The spike responses were thus reduced for low-intensity sounds. We then address the question whether adaptation leads to an improvement of the signal's representation and compare the experimental results with the predictions of two competing hypotheses: infomax, which predicts that information conveyed about the entire signal range should be maximized, and selective coding, which predicts that “foreground” signals should be enhanced while “background” signals should be selectively suppressed. We test how adaptation changes the input–response curve when presenting signals with two or three peaks in their amplitude distributions, for which selective coding and infomax predict conflicting changes. By means of Bayesian data analysis, we quantify the shifts of the measured response curves and also find a slight reduction of their slopes. These decreases in slopes are smaller, and the absolute response thresholds are higher than those predicted by infomax. Most remarkably, and in contrast to the infomax principle, adaptation actually reduces the amount of encoded information when considering the whole range of input signals. The response curve changes are also not consistent with the selective coding hypothesis, because the amount of information conveyed about the loudest part of the signal does not increase as predicted but remains nearly constant. Less information is transmitted about signals with lower intensity.

## Author Summary

Sensory systems have the ability to adapt to changes in the environment. In a quiet room, the nervous system is very responsive, so that even a whisper can be easily understood. In contrast, the perceived loudness on a crowded street will be reduced to prevent an overload of the nervous system. Two different hypotheses have been proposed to explain how the nervous system achieves this adaptation. According to one idea, all present sensory signals are equally enhanced, so that the whole range of input signals is reliably represented. On the other hand, the aim of the nervous system may be to extract the most important parts of the acoustic signal, for example, an approaching car, and thus abolish the irrelevant rest. To address which of these two principles is implemented in the auditory system of the cricket, we investigated the responses of a single auditory neuron, called interneuron AN2, to different sound signals. We found that adaptation actually reduces the amount of encoded information when considering the whole range of input signals. However, the changes were also not in agreement with the idea that only the most important signal is transmitted, because the amount of information conveyed about the loudest part of the signal does not increase. Thus, we here report the unusual case of a reduction of information transfer by adaptation, while in most other systems reported of so far adaptation actually enhances coding of sensory information.

## Introduction

Efficient encoding of natural signals is one of the major tasks sensory pathways have to accomplish. In order to do this, neural representation should be matched to the relevant part of incoming signals. Statistical properties of incoming signals are highly variable in a natural environment (e.g., the mean light level changes dramatically from a sunny region to a dark forest) but are mostly changing slowly over time [1]. Since the neural representation in sensory cells is limited to a certain range and resolution, the principle of efficient coding suggests that the nervous system should continually adapt its responses to changing statistical properties of the stimuli [2]. Firing rate adaptation changes the input-response curves of neurons in sensory pathways and has been shown to provide a mechanism for the adjustment of the encoding scheme in multiple systems [3]–[9]. How the input-response curve is altered in response to a given stimulus should depend on what the relevant information is in the given context.

Here, we want to explore the response properties of a single cell (AN2) in the auditory pathway of crickets and test for two different principles that have been proposed to underlie adaptation of the input-response curve: the principle of maximum information preservation (infomax) [10] and that of selective coding [11]. The AN2 neuron provides an ideal model for studying the computational principles underlying adaptation, since (1) it receives direct input from auditory receptors and local interneurons at the first processing level [12], (2) on present evidence, it constitutes the only ascending representation of the auditory environment in the high frequency channel and thus a bottleneck for information transmission to higher centers [13]–[15], and (3) it has a clear behavioral role because it is intimately involved in evasive behavior in response to ultrasonic signals [16]–[18]. Several time constants of adaptation in the range from below 100 ms to several seconds are known for the receptor cells [19], local interneurons [20], and the ascending neurons [21],[22] in this model system. Since auditory processing at the stage of the AN2 neuron is mainly feed-forward, adaptation is likely driven by the stimulus only rather than by task-dependent top-down processes.

**Figure 1:**
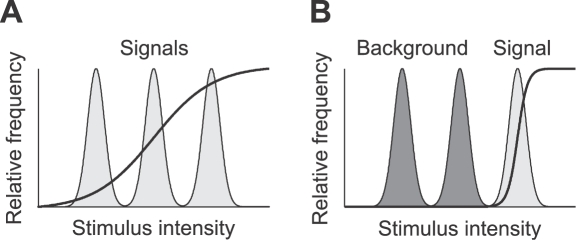
Optimal sigmoidal stimulus response curves (solid lines) for a stimulus distribution consisting of three peaks (shaded areas) as predicted by two coding hypotheses. (A) Infomax: the dynamic range of the adapted response curve covers the whole range of input signals. Note that the optimal *sigmoidal* response curve is shown; generic optimal transmission would be attained by a response curve that has a derivative proportional to the local stimulus distribution. Such a response curve would be steep within peaks of the stimulus distribution and much flatter in between, thus it would be more staircase-like. (B) Selective coding: the response function optimally represents the most intense signal (light gray) whereas other signals (dark gray) are suppressed.

The above mentioned principles lead to conflicting hypotheses about changes of the input-response curve when more than one ‘signal’ is present in an environment (Figure 1). Following the infomax principle, the input–output transformation (the neuronal response curve) should maximize the information transmission between the neural representation and the stimulus. The optimal response curve depends on the statistical properties of the input signals, but internal noise and constraints on the possible changes limit the amount of information that can be conveyed.

The infomax principle leads to the theoretical result that the derivative of the response curve should be proportional to the probability distribution of the stimuli, so that all available signals in a given environment are represented and every possible output rate occurs with equal probability. Laughlin [23] tested this prediction and showed that contrast response curves in the fly visual system are matched to the statistics of natural images in order to maximize information transmission. Similar results have been reported for contrast response curves of retinal and LGN neurons in cat and monkey [24]. Information maximization can explain retinal coding in the spatial, temporal and chromatic domain [25].

A consequence of the infomax principle is that a change in the statistics of the sensory input must be compensated by a change in the input–response curve. Experimental evidence from the motion-sensitive H1 neuron in the fly supports this hypothesis: this neuron adapts its response curve to changing statistics of stimuli on several time scales [4],[6], in a way which is compatible with the infomax prediction. Experiments have also shown that adaptation enhances information transmission in visual cortex [26]. Sharpee et al. estimated neural filters for the responses to natural inputs and to noise inputs matched for luminance and contrast, showing that neural filters adaptively changed with higher order statistics of input signals, so as to increase the mutual information between stimulus and neural response. Theoretical work also suggested that contrast adaptation in the mammalian visual system [3], [9], [27]–[29] can be understood as a consequence of the infomax principle [30],[31]. However, it is difficult to quantify the role of adaptation in enhancing coding efficiency at higher stages in vertebrate sensory pathways since in these, coding is distributed among large populations of neurons and their responses are modulated by the activity of other neural populations or brain areas. Thus, simple sensory networks of invertebrates, whose representation are not heavily influenced by feedback signals, may provide a more suitable model to understand the principles lying behind sensory adaptation.

An alternative principle that may underlie adaptation is selective coding (or ‘background suppression’), a form of temporal inhibition in which a loud sound suppresses the response to subsequent sounds. This could serve to segregate a single, most important signal from other signals or background noise. It has been shown that an auditory interneuron (ON1) represents mainly the louder part of a stimulus with a bimodal intensity distribution [20]. Calcium aggregation in the omega neuron is a possible mechanism underlying this background suppression [11],[32]. Similar findings have been made in bushcrickets: while multiple songs in choruses of singing males are present, only the most intense song was found to be represented in the auditory pathway [33]. These previous studies, however, address the phenomenon only qualitatively and not under the viewpoint of an encoding scheme and information transfer. Segregation of different auditory objects into different channels has also been studied in vertebrate hearing [34]–[36]. In vertebrates, however, modulation of carrier frequency is assumed to play a crucial role in this stream segregation [37], complicating a detailed analysis. Information conveyed by carrier frequency modulation is very limited in crickets, as they possess only two broadly tuned frequency channels, one in the range around the carrier frequency of the calling songs (∼5 kHz) and another one mainly for frequencies above 12 kHz. Thus, crickets provide an ideal model system to study object-background segregation—in this simple auditory system, for a given frequency range, an auditory object can simply be seen as the loudest peak in the entire stimulus distribution.

The questions we sought to answer were: How does the neural response curve adapt to the statistics of the acoustic environment? Can this sensory system be characterized as a communication channel optimized for coding the inputs such that as much information as possible is preserved (infomax principle)? Or does the system perform a preprocessing that leads to a high fidelity representation of only the loudest part of the stimuli (selective coding)?

To address these questions, we measured the neural response curve of AN2 neurons after adaptation to sound stimuli with either two or three peaks in their intensity distribution, depicted in Figure 1. The two principles studied here predict conflicting changes of the form of the input-response curve when presenting a stimulus composed of more than one signal. Optimal selective coding should lead to a shift of the response curve in a way that only the peak with the highest intensity is represented (Figure 1B). If infomax is the underlying principle, adaptation pursues the objective to maximize the information that the neuron's output conveys about its sensory input. Adaptation should thus change the response curve in a way that the whole stimulus range is encoded reliably (Figure 1A).

Firing rate adaptation can change the stimulus–response curve basically in two ways [38]: shifting the threshold to larger intensities and changing the slope of the curve. We first compare the experimentally observed changes in the slope and the shift in response curves to the optimal changes predicted by the two competing hypotheses. Differences between model prediction and data, however, do not necessarily imply that a particular hypothesis is unlikely to be true, because additional constraints may limit the potential of tuning curve changes. Therefore, in a second step, we calculate the mutual information between the sensory input and the neuronal response using the measured response curves. The infomax principle predicts that the mutual information between a particular stimulus distribution and the response should be highest for the response curve that is adapted to the stimulus distribution. The response curve adapted to the stimulus with three peaks should encode the three-peak stimulus better than the response curve adapted to the stimulus with two peaks. Selective coding, on the other hand, predicts, that the mutual information should decrease for the ‘background’ signals and should increase for the most intense peak.

## Methods

### Animal Preparation

Crickets of the species *T. oceanicus* and *T. leo* were used in the experiments to characterize the time course of adaptation. For the experiments with the multimodal stimuli (cf. Methods, Stimulus protocols), mainly *T. leo* individuals were used. All animals were laboratory reared. For preparation, both pairs of wings and the meso- and metathoracic legs were removed. The animal was fixed ventral side up to a small platform and the prothoracic legs with the ears were waxed to pins at the coxae and the tarsi in a normal walking position. Ascending and descending connectives from the prothoracic ganglion were cut in order to reduce neuronal background activity. See [12] for a more detailed description.

### Recordings and Acoustical Stimulation

**Figure 2:**
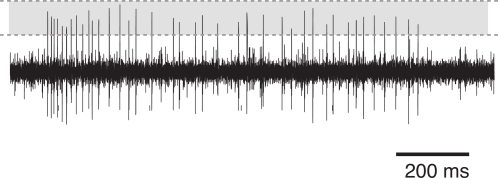
Typical recording trace from a cricket AN2 neuron (*T. oceanicus*). The figure shows the voltage trace during constant stimulation (duration 1 s) with a sinusoidal tone of 16 kHz frequency. The shaded area depicts the spike detection window, bounded by the lower and upper threshold.

Two extra-cellular hook-electrodes were made from tungsten wire and placed in parallel around one of the two connectives ascending from the prothoracic ganglion. These connectives contain the axon of the ascending interneuron we wanted to record from (AN2). Vaseline was placed around connectives and hooks in order to isolate the electrodes electrically and keep the connective from drying out. The voltage trace was amplified differentially (npi, EXT-10C, Tamm, Germany) and bandpass-filtered with cut-off frequencies of 300 Hz and 3 kHz (npi, DPA 2F). The trace was then digitized at 20 kHz sampling rate (National Instruments, PCI-6014, Austin, TX) and stored to the hard disk of a personal computer. Spikes of the AN2 were detected on the basis of the amplitude peaks of the voltage trace using custom Software (MATLAB 7, The MathWorks, Natick, MA). Figure 2 shows an example recording and the spike detection window.

The recording set-up was lined with sound-absorbing foam to reduce echoes. Acoustic stimuli were presented through a loudspeaker positioned ipsilaterally to the recorded connective at a distance of 36 cm. The main input to the AN2 neuron comes from receptors ipsilateral to the connective that holds its axon. Stimuli were presented by analog multiplication of a generated 16 kHz sine wave (Voltacraft, FG-506, Hirschau, Germany) with an amplitude modulation envelope that was generated by the personal computer at 10 kHz sampling rate (National Instruments, PCI-6014, Austin, TX). Following this, the signal was attenuated using a programmable attenuator (Tucker-Davis, PA5, Gainesville, FL) and amplified by an audio amplifier (Blaupunkt, GTA 2100B, Hildesheim, Germany). Attenuation of the signal was calibrated using a Bruel & Kjaer microphone (type 2231, Bremen, Germany).

### Stimulus Protocols

**Figure 3:**
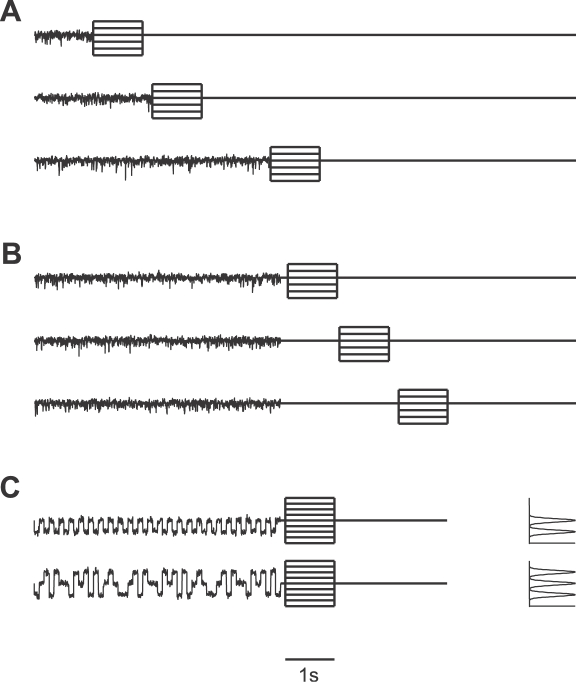
Summary of the experimental protocols. (A) Adaptation protocol. Amplitude-modulated noise signals (adapting stimuli with 0 dB average relative intensity) of variable duration (from 75 ms to 4800 ms) are followed by a test stimulus (16 kHz sinusoidal tone) with a duration of 1000 ms and a relative intensity ranging from −9 dB to +6 dB (several test stimuli are plotted on top of each other). (B) Recovery protocol. Amplitude-modulated noise signals (adapting stimuli) of 5 second duration are followed by a pause of variable length (from 75 ms to 4800 ms) and a test stimulus as in (A). (C) Adaptation protocol for amplitude-modulated noise stimuli drawn from a bimodal and a trimodal distribution (the corresponding amplitude distributions are shown in the right panel). Relative intensities of the test stimuli range from −6 dB to +6 dB.

Figure 3 shows the protocol used for characterizing the adaptation process in the ascending AN2 neuron. The different ensembles of auditory stimuli consist of an adapting stimulus, a silent interval, and a test stimulus. The intensity of the adapting stimulus was adjusted in the beginning of the recording depending on the response strength of the neuron. Normally, the base line intensity of the adapting stimulus had a sound pressure level of 84 dB or 87 dB. With the term *relative intensity* we refer to the stimulus intensity relative to this base line intensity. Adapting stimuli are 16 kHz signals that were amplitude-modulated by bandpass-filtered Gaussian white noise with 100 Hz cut-off frequency. The Gaussian noise had a variance σ^2^ = 1.38 dB^2^ and a mean relative intensity μ = 0 dB. Test stimuli were pure sinusoidal tones with a frequency of 16 kHz. To characterize adaptation, we used adapting stimuli with durations 75 ms, 150 ms, 300 ms, 600 ms, 1200 ms, 2400 ms, and 4800 ms (Figure 3A). For testing recovery from adaptation, the stimuli had a 5 s adaptation phase followed by pauses of varying durations from 75 ms to 4800 ms (Figure 3B).

Motivated by the competing coding hypotheses, we wanted to examine the consequences of adaptation to different ensembles of auditory stimuli demanding different changes in the stimulus-response curve. We designed multimodal noise-like stimuli whose amplitude distribution had two or three modes, mimicking auditory scenes with multiple signals. The amplitude distribution of the bimodal stimulus is composed of two Gaussian distributions, with mean relative intensity μ_1_ = −3 dB, μ_2_ = 0 dB and variance σ^2^ = 0.2 dB^2^. The trimodal stimulus has an additional peak modeled by a third Gaussian distribution with mean μ_3_ = +3 dB and σ^2^ = 0.2 dB^2^. An example of these stimuli is shown in Figure 3C, together with the respective amplitude distributions. The adaptation time was 5 s in these experiments and the silent interval before the test stimulus was 100 ms.

In all experiments, the intensity response curves were determined by sinusoidal test stimuli with a frequency of 16 kHz and duration of 1 s, following the respective adaptation stimulus. The relative intensities of the test stimuli were −9 dB to +6 dB. Each stimulus was presented at least five times.

### Bayesian Data Analysis

**Figure 4:**
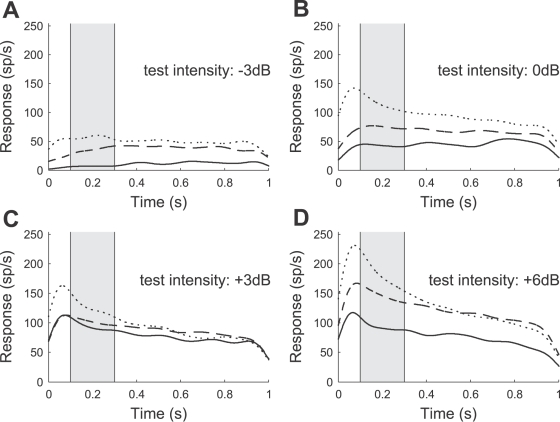
Representative examples of the neural response (AN2 neuron from a *T. leo*) after adaptation to noise stimuli of duration 75 ms (dotted line), 600 ms (dashed line), and 4800 ms (solid line). (A–D) Responses (spike rates) during a test stimulus of 1 s duration (cf. protocol of Figure 3A). Relative intensities of the test stimuli range from −3 dB (A) to +6 dB (D); the average relative intensity of the adapting stimulus was 0 dB. Each stimulus was presented 5 times and the recorded spike trains (1 ms resolution) were convolved with a Gaussian kernel (σ = 50 ms). The instantaneous spike rates were estimated by averaging over the 5 repetitions. The increase of the estimated rate during the first 50 ms is an artifact introduced by filtering the neural response with the Gaussian kernel. Note that the onset latency of the AN2 neuron is in the range of 15 to 18 ms. The spike counts during the sample period (shaded) from 100 ms to 300 ms are used to construct neural response curves.

**Figure 5:**
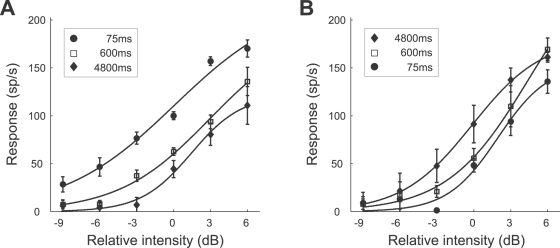
Representative example of response curves for different adaptation (A) and recovery times (B) (cf. protocols of Figure 3A and 3B). The average relative intensity of the adapting stimulus was 0 dB. Symbols denote the average spike counts during the sample period (cf. Figure 4) for different test intensities. Solid lines indicate the expected response curve, i.e., the response curve with the set of parameters with the mean value of the posterior distribution (see Methods, Bayesian data analysis). Each stimulus protocol was repeated 5 times (the error bars indicate the standard deviation). The data shown was obtained from a *T. leo* (the same preparation as used in Figure 4).

We constructed intensity-response curves to quantify the neural response, as shown in Figure 4 and Figure 5. Therefore, we used the spike count in a 200 ms time window beginning 100 ms after test stimulus onset. The window was chosen such that the influence of the fast adaptation process (time constant of about 40 ms, similar to the one described for the AN1 neuron by Benda and Hennig [21]) is minimized. In the context of this separation of time scales, we are interested only in the coding of slower stimulus dynamics. Hence we consider responses to unmodulated test stimuli and measured spike counts within a 200 ms—rather than a short—time window.

A common methodology to construct neuronal response curves is repeating a single experimental condition several times and then computing the mean of the observed spike counts and their variance. In a second step, a parametric model is fit to these data, typically using least-square approximation. Often it is interesting, however, how the parameters of the response curve change with different experimental conditions, but the confidence intervals for the model parameters and tests for the significance of parameter changes are difficult to establish with traditional statistical methods.

Here, we use a Bayesian analysis [39],[40], to account for the statistics of each trial to estimate the parameters accurately and to quantify the confidence limits of the parameter estimation. The method allows estimating the full probability distribution of the response curve parameters rather than only the mean value as with traditional methods. Similar techniques have been applied successfully to the analysis of intracellular membrane potential recordings [41].

#### Modeling the sound-pressure-level to spike-count relation

The analysis is based on the assumption that spikes are Poisson-distributed and that individual trials are independent of each other (i.e., their joint probability is equal to the product of their individual probabilities).

Let *x_i_* denote the *i*th out of *m* stimulus intensities and *n_i_* the number of times a stimulus with this intensity is presented. The corresponding number of spikes from an AN2 neuron is denoted by *y_i,j_*, where *j* is the *j*th out of the *n_i_* repetitions. If spikes are Poisson distributed, we obtain
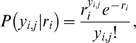
(1)where *r_i_* is the average spike count underlying the neuron's response at the *i*th stimulus intensity. For a set *y_i_* = (*y_i_*_,1_, …, *y_i,ni_*) of spike counts of *n_i_* independent and identically distributed observations, the likelihood *P*(*y_i_*|*r_i_*) of *r_i_* being the underlying average spike count becomes
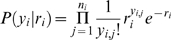
(2)

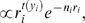
(3)where the likelihood function is determined, up to a constant factor, by the sufficient statistic
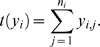
(4)


We assume a sigmoid response curve, relating stimulus intensity to spike counts as
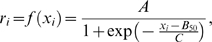
(5)where *r_i_* is the underlying average spike count of the neuron, *x_i_* is the stimulus intensity, *A* is maximum response of the cell, *B*_50_ is the stimulus intensity at 50% of maximum response, and *C* is a slope factor. Inserting this relationship into Equation 3, we obtain the likelihood *P*(*y_i_*|*r_i_*) in terms of the response curve parameters *A*, *B*_50_, and *C*:
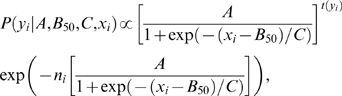
(6)Let *y* = (*y*_1_, …, *y_m_*) be the set of responses to stimuli with different intensities *x_i_*, where *I* = 1…*m*. Applying Bayes' rule we obtain the joint posterior distribution
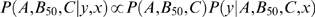
(7)

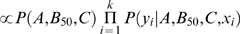
(8)of the parameters *A*, *B*_50_, and *C*, given the observations, where *P*(*A*, *B*_50_, *C*) is the prior distribution of the response curve parameters *A*, *B*_50_, *C*. In the following, we will use a noninformative, uniform prior distribution *P*(*A*, *B*_50_, *C*) = constant.

#### Calculating the joint posterior distribution

Following [39], the posterior was calculated for a range of *A*, *B*_50_, and *C* values using a grid of 200×200×200 points and normalized across this grid. Initially a large parameter space was sampled (e.g., values for the parameter *A* in the range from 0 to three times the maximum observed spike count of the neuron) that was narrowed to allow finer sampling in the region of non-zero posterior values. To simplify further analysis we then draw 10000 independent and identically distributed random samples (*A_i_*, *B*_50,*i*_, *C_i_*), where *I* = 1 … 10000 from the joint posterior probability distribution. From these samples, we can estimate the posterior distribution of any quantity of interest, e.g., the posterior distribution of the response curve parameters ‘location’ *B*_50_ or of the ‘slope’ at half of the maximum response:
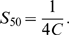
(9)The slope *S*_50_ does not depend on the maximum response *A*, in order to be able to compare the response curve slopes from different neurons (i.e., for calculating the slope the neural responses are normalized to the interval between 0 and 1).

To summarize the results for all the recorded AN 2 cells, we combined the samples from the posterior distributions of individual cells to obtain a ‘combined posterior distribution’ (assuming independence of individual experiments).

When reporting experimental results, we will in most cases characterize the corresponding posterior distributions by their mean values (i.e., the expected values of the parameters, given the data). We also use these expected parameter values to illustrate the estimated sigmoid response curve. In most cases, the expected parameters and the parameters with the maximum posterior probability (i.e., the maximum a posteriori estimate) had very similar values.

#### Significance testing

Consider one of the parameters of interest, e.g., *B*_50_, and its posterior distributions 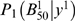 and 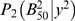, for two stimulus conditions 1 and 2. Bayesian analysis provides us with samples from these distributions *P*_1_ and *P*_2_. To determine if 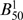 is significantly different from 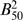, we calculate the posterior distribution *P_d_* of the difference 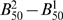. This is done by repeatedly taking one sample *b*_1_ from the distribution 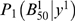 and one sample *b*_2_ from 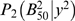 and calculating the difference *b*_2_–*b*_1_, giving one sample from the distribution *P_d_*.

To determine a significant difference, we calculate the 95% posterior interval [*i*_1_,*i*_2_] of *P_d_*, defined as the range of values above and below which lie 2.5% of the samples. The values *i*_1_ and *i*_2_ can be directly estimated from the samples: *i*_1_ corresponds to the 2.5th and *i*_2_ to the 97.5th percentile. If the 95% posterior interval of *P_d_* includes zero, the difference between 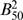 and 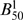 is not statistically significant. On the other hand, if the 95% posterior interval excludes zero we regard the difference as significant. To test if an estimated parameter is significantly larger (smaller) than a certain value *x*, we calculate the right-tailed (left-tailed) posterior interval. If the right-tailed (left-tailed) posterior interval excludes the value *x*, i.e., less than 5% of the corresponding samples are smaller (larger) than *x*, we regard the parameter significantly larger (smaller) than *x*.

#### Time course of adaptation

We used a single exponential decay model to characterize the time course of adaptation
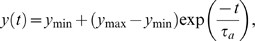
(10)where *y*(*t*) is the neural response at time *t*, *y*_min_, and *y*_max_ are minimum and maximum response, and *τ_a_* is the decay time constant. A similar single exponential model
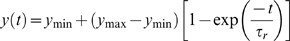
(11)is used for describing the recovery from adaptation, where *τ_r_* is the recovery time constant. Using the Bayesian approach, we calculate the posterior densities of the parameters of Equations 10 and 11 in a similar manner as for the sigmoid response curve (Equation 5).

### Numerical Estimation of Mutual Information

The mutual information *I*[*Y*;*X*] between the sensory signal *X* and the neural response *Y* specifies how much information is conveyed on average about all possible signals. In order to compute the mutual information numerically, taking into account the influence of discrete, Poisson distributed spike counts, we first construct the joint probability distribution
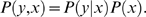
(12)For each stimulus intensity *x_i_*, we calculated the corresponding average spike count *r_i_* using Equation 5. The distribution *P*(*y*|*x* = *x_i_*) is then given by a Poisson distribution with mean *r_i_* (Equation 1). For all simulations, the stimulus *X* was discretized into bins of size 0.01 dB. At this resolution, the results did not depend on the bin size.

To measure the information that is associated with specific sensory signals, we define the stimulus-specific information [42],[43]:
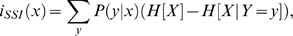
(13)where *H*[*X*] = −Σ*_x_ P*(*x*) log_2_ *P*(*x*) is the entropy of the sensory signal *X*, and the conditional entropy of a particular response *y* is given by *H*[*X*|*Y* = *y*] = −Σ*_x_ P*(*x*|*y*) log_2_ *P*(*x*|*y*). Stimulus-specific information can be interpreted as the average reduction of uncertainty about the sensory signal gained from one measurement given the stimulus *x*. Taking the weighted average over the stimulus-specific information for all possible signals we obtain the mutual information between stimulus and response:
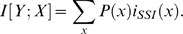
(14)To determine the information associated with a certain stimulus range [*x*_1_,*x*_2_], we evaluate the sum in Equation 14 from *x*_1_ to *x*_2_.

## Results

### Time Course of Adaptation

We first studied the effects of prolonged auditory stimulation in recordings of 6 AN2 neurons of *T. oceanicus* and 7 AN2 neurons of *T. leo*. Previously, an adaptation process operating on a time scale of 40 ms had been characterized for the AN1 neuron [21]. Here, we investigate whether adaptation also occurs on a slower time-scale, better matched to changes in the acoustic environments.

#### Adaptation and recovery

We recorded the responses of AN2 neurons to test stimuli of different intensities, after adaptation to noise stimuli of varying duration (see Methods, Stimulus protocols). A typical example for the neural responses of an AN2 cell of *T. leo* is shown in Figure 4. The spike rates after an adaptation period of 4800 ms are always lower than the corresponding responses after 600 ms and 75 ms adaptation time. Responses declined with prolonged stimulation during the test interval for the applied intensities that were higher than the intensities of the adapting stimuli (Figure 4C and 4D), a phenomenon which we observed in all the recorded cells. The rapid initial change, which is most pronounced for high intensities of the test stimulus (Figure 4C and 4D) is caused by the fast firing-rate adaptation (similar to the adaptation in the AN1 neuron [21]). To minimize an influence of the fast and the slow adaptation occurring during test, only spikes occurring between 100 ms and 300 ms after test stimulus onset were used for further analysis (see Methods, Bayesian data analysis). Figure 5A shows the stimulus response curves constructed from the spike counts within the abovementioned interval. Prolonged stimulation shifted the stimulus-response curves towards higher stimulus intensities. In the example shown, adapting for 4800 ms virtually eliminated the response to low relative intensities from −9 dB to −3 dB. Adaptation changes the range of relative intensities over which the cell responds, but has little effect on the maximal firing rate. Figure 5B shows data from the same cell when using stimuli for testing the recovery from adaptation (see Methods, Stimulus protocols). Adapting stimuli were always 5 s long, followed by a silent interval of varying duration and a test stimulus. After a recovery period of 4800 ms the neuron has almost recovered its state prior to adaptation. Hence, adaptation and recovery from adaptation operate on a similar time scale.

#### Time constants of adaptation

**Figure 6:**
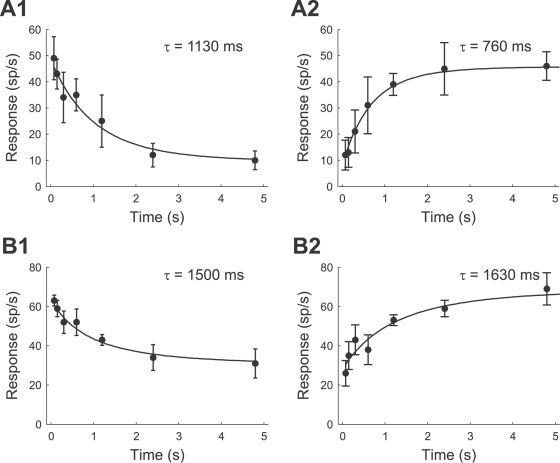
Time course of adaptation and recovery of a *T. leo* cell (A1,A2) and of a *T. oceanicus* cell (B1,B2). The response to the test stimulus is plotted against the duration of the adapting stimulus (A1,B1) and the delay between the adapting and the test stimulus (A2,B2). Displayed are the average spike counts in the 200 ms time window of the test stimulus (cf. Figure 4). The intensity of the test stimulus was equal to the average intensity of the adapting stimulus (0 dB relative intensity). The error bars denote the standard deviation. Solid lines indicate the exponential function with the set of parameters with the highest value of the posterior distribution (see Methods, Bayesian data analysis).

To quantify the time course of adaptation and recovery we analyzed the neural responses to test stimuli that had the same relative intensity as the adapting stimuli (0 dB). Additional cells were recorded with a reduced version of the stimulus protocol that only included these 0 dB test stimuli (the total number of cells available for each species and each stimulus protocol is stated in Table 1). In order to determine the adaptation and recovery time constants *τ_a_* and *τ_r_*, we fitted an exponential decay model to the neural responses (see Methods, Bayesian data analysis). Figure 6A and 6B show examples of recorded data and exponential fits for a *T. oceanicus* and a *T. leo* cell. Both time constants lie in the range of 1 second for both of these cells. This is considerably longer than the short-term firing rate adaptation, which operates on a time scale of 40 ms.

**Table 1:** Summary of the adaptation (*τ_a_*) and recovery (*τ_r_*) time constants for the *T. oceanicus* and *T. leo* AN2 cells.

Species	Adaptation			Recovery from adaptation		
	*τ_a_* (ms)	SD (ms)	*n*	*τ_r_* (ms)	SD (ms)	*n*
*T. oceanicus*	1202	558	6	1947	1155	6
*T. leo*	1828	939	9	1674	582	11

See Figure 3 for the adaptation protocols. SD is the standard deviation across the *n* cells.

**Figure 7:**
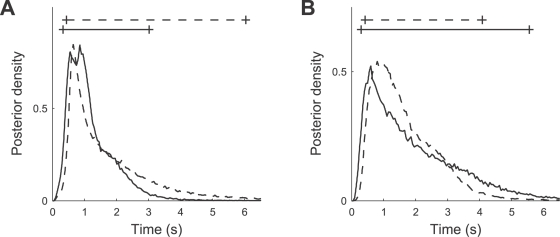
Combined posterior distribution (cf. Methods, Bayesian data analysis) of the adaptation time constants *τ_a_* (A) and the recovery time constants *τ_r_* (B) for the *T. oceanicus* (solid line) and *T. leo* (dotted line) AN2 cells. Solid (dotted) lines on top of the figures depict the 95% posterior intervals.

The values of the adaptation and recovery time constants are summarized in Table 1 for both species; additionally, Figure 7 shows the combined posterior distributions (cf. Methods, Bayesian data analysis). Comparing the time constants between *T. oceanicus* and a *T. leo* cells, we did not find significant differences, as reflected by the overlapping 95% posterior intervals in Figure 7. Furthermore, adaptation and recovery time constants have similar values.

We conclude that the neuronal responsivity of AN2 neurons is affected significantly by adaptation and that the adaptation process operates on a time scale of seconds. The primary effect is a change in the range of stimulus intensities over which the cell responds. To put to test our hypothesis that this adaptation serves for adjusting the stimulus–response curve to the current acoustic environment, we first formalize the infomax and selective coding principle and then assess the experimentally observed response curve changes.

### Quantitative Predictions of the Coding Hypotheses

The infomax principle and the selective coding hypothesis both predict how the neural response curve should optimally change in response to a change in the statistics of the environment. In order to assess the response curve changes quantitatively, we first compute the parameters of the optimal response curve under either hypothesis as well as the mutual information between stimulus and neural response.

#### Infomax principle

If infomax [10],[25],[30] is the underlying principle, adaptation pursues the objective to maximize the information that the neuron's output conveys about its sensory input. Formally, the goal is maximizing the mutual information *I [R; X]* between the sensory sound signal *X* and the neuronal output firing rate *R* as a function of the response curve parameters. This is achieved by maximizing the output entropy *H [R]* while minimizing the uncertainty *H [R|X]* of the output once the input is fixed
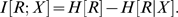
(15)We first computed the optimal response curve parameters for a given signal distribution and a sigmoid response function analytically, assuming additive noise. Next, we estimated the mutual information numerically in order to account for multiplicative (Poisson) noise. If we assume only additive noise, with a probability distribution *P*(*n*), the mutual information can be written as [44],[45]

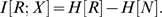
(16)Maximization of the mutual information is then equivalent to the maximization of the entropy of the output distribution, because the noise entropy *H*[*N*] does not depend on the input–output mapping, i.e., the neural response curve *r*(*x*). Thus, we have to maximize
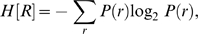
(17)where the sum goes over all possible discrete response levels *r* (here, the spike counts in a 200 ms window; cf. Bayesian data analysis). Formally, we can treat the response as a continuous variable [25], i.e., as firing rate, and using the relationship between differential and discrete entropy we approximate the sum by an integral [46]:
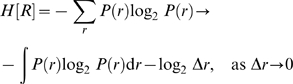
(18)Here, Δ*r* is the limit on the resolution with which the firing rate can be measured (the length of the bins of the discrete response levels). Note that in the limit Δ*r*→0 the entropy *H*[*R*] diverges (i.e., the information capacity of a continuous variable is unlimited). In the case Δ*r*→0 and in the absence of noise the sensory signal *X* could be recovered perfectly from the firing rate *R* and thus any set of response curve parameters would be ‘optimal’. However, if we assume a finite maximum of the response curve the additive noise provides a resolution scale on the output and we can ask for an optimal response curve *f*(*x*). In the low-noise limit we obtain [45]

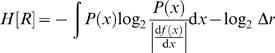
(19)

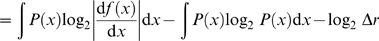
(20)Since the second term of Equation 20 only depends on the signal distribution and the third term only depends on the resolution Δ*r*, they are constant, and we have to maximize:
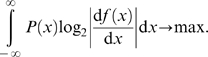
(21)To compare how well a given sensory signal *X* with distribution *P*(*x*) is encoded by response functions with different parameterizations (*r_I_* and *r_II_*), we compute the difference in mutual information Δ*I*. Under the assumption of small additive noise and for a fixed resolution Δ*r*, this difference in mutual information is given by
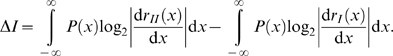
(22)


**Figure 8:**
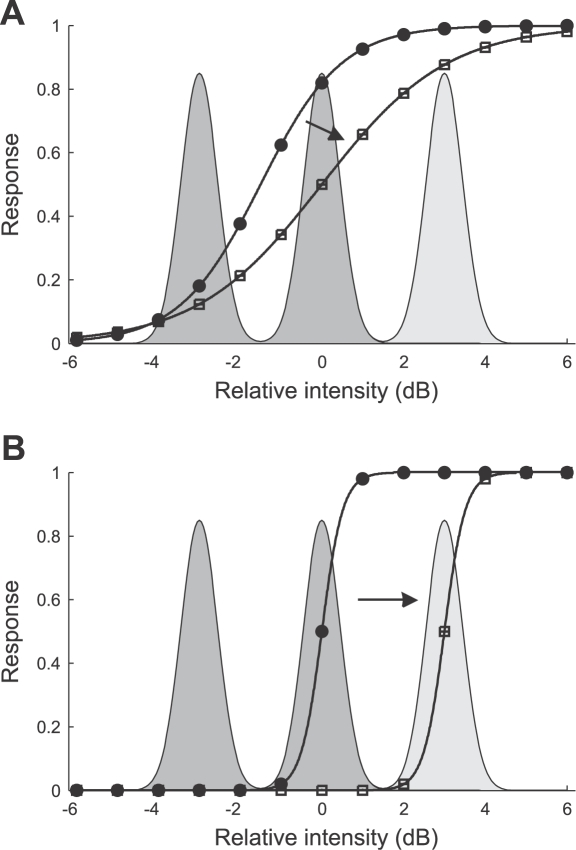
Optimal response curves for the bimodal (circles) and trimodal (squares) stimulus distribution predicted by the infomax principle (A) and the selective coding hypothesis (B). The figures show the predicted relationship between the response variable (spike rate) and the stimulus intensity. The Gaussian curves depict the probability distributions of stimulus intensity, where the dark shaded areas under the curve denote the bimodal stimulus distribution and the light shaded area under the curve the additional peak of the trimodal stimulus distribution (cf. Figure 1).

Assuming the sigmoid transfer function of Equation 5 and the bimodal or trimodal stimulus distribution (see Methods, Stimulus protocols), we obtain the optimal values for the response curve parameters *A*, *B*_50_, and *C* using Equation 21; the optimal value for the slope *S*_50_ is then computed using Equation 9. Figure 8A shows the predicted response curves for both stimulus distributions, under the assumption that the response curve parameters *B_50_* and *S_50_* can be optimally adjusted. The optimal value for *B*_50_ is −1.50 dB for the bimodally distributed stimulus and 0.00 dB for the trimodally distributed stimulus, corresponding to a response curve shift of +1.50 dB. To cover the whole stimulus range, the slope should decrease for the trimodally distributed stimulus compared to the bimodally distributed stimulus by −35.3%, from 0.25 dB^−1^ to 0.16 dB^−1^. If we assume that the neural system can only adjust *B*_50_ and the slope *S*_50_ is constant, the infomax principle would still predict a shift of the response curve of 1.50 dB. Evaluating Equation 22, the infomax principle then predicts that information transmitted about the trimodal stimulus will improve by 0.61 bit for the trimodally adapted response curve compared to the bimodally adapted response curve. Note that this calculation involves the assumption of low additive noise and a fine resolution Δ*r*. Therefore, we also calculated the predicted increase in information transmission numerically (see Methods, Numerical estimation of mutual information), assuming discrete, Poisson distributed spike counts. In this case, the improvement in information transmission depends on the maximum spike count, defined by the response curve parameter *A*. For the experimentally observed maximum spike counts in the range of 20 spikes to 55 spikes we obtain an increase from 0.12 bit (20 spikes) to 0.25 bit (55 spikes).

#### Selective coding

Selective coding is a concept which is less well defined than the infomax principle, because it involves an assumption about the ‘signal’ vs. the ‘background’ part of a complex stimulus. In the following we assume, that the loudest signals of artificial environments, i.e., the ‘loudest’ Gaussian distributions of the multimodal stimulus distributions (see Figure 8B) are encoded in an optimal way while the other (‘background’) signals are suppressed. To compute the optimal response curve for the bimodal (trimodal) stimulus distribution the objective is to maximize Equation 21 taking the Gaussian with μ = 0 dB (μ = 3 dB) as the ‘signal’ part. Figure 8B shows the predicted response curves when we assume that the loudest signal should be encoded reliably and other signals should be suppressed. The predicted difference between the response curve optimized for the bimodal and trimodal stimulus is a shift by 3.00 dB (from *B*_50_ = 0.00 dB to *B*_50_ = 3.00 dB). The slope *S*_50_ does not change and remains at 0.98 dB^−1^.

Surely, the response curves shown in Figure 8B are idealized but they illustrate the consequences of the selective coding vs. the infomax principles: according to the infomax principle, information transmission is optimized for the whole stimulus range, while selective coding implies a selective enhancement or a selective suppression of the transmitted information for certain kinds of stimuli. To quantify this selective stimulus encoding, we calculated the information associated with parts of the stimulus range numerically (see Methods, Numerical estimation of mutual information), for the trimodal stimulus and the predicted response curves (Figure 8B). The maximum responses *A* were determined by the experimental data (from 20 spikes to 55 spikes), and noise was Poisson distributed. Next, the mutual information is evaluated for the stimulus range of −4.5 dB to 1.5 dB (background signals) and for the range of 1.5 dB to 4.5 dB (loudest signal) using Equation 14. We find, that the information transmitted about the loudest peak of the trimodal stimulus distribution is enhanced by 0.51 bit (0.70 bit) for *A* = 20 (55) spikes when using the optimal response curve for the trimodal stimulus, while at the same time less information (−0.811 bit for *A* = 20 spikes; −1.07 bit for *A* = 55 spikes) is conveyed about the first and second peak.

#### Optimality vs. improvement

The infomax and selective coding hypotheses (as specified above) both make quantitative predictions of the optimal response curve parameters and how these parameters should change to optimally adjust the response curve to a changed stimulus statistics. If selective coding is the underlying principle, the response curve should be steeper than for the infomax principle. When the environment changes from a bimodal to a trimodal input distribution, selective coding predicts a large shift of the response curve towards higher stimulus amplitudes, whereas the slope should remain constant. The infomax principle, on the other hand, predicts a less pronounced shift and a decrease in slope.

However, architectural constraints might prevent the AN2 neuron from achieving the theoretically optimal response curve. It is conceivable that, for example, the neural gain cannot increase such that the slope of the stimulus-response curve would be optimal for encoding only the loudest peak of the stimulus distribution as required by ‘optimal’ selective coding. How can we quantify the improvement in neural coding according to the one or the other hypothesis without requiring optimality?

Both the infomax principle and selective coding also predict characteristic changes in mutual information between the stimulus and the response for a change from the bimodal to the trimodal environment. Following the infomax principle, the associated response curve change leads to an increase in mutual information. Selective coding, on the other hand, leads to a selective decrease (increase) of the mutual information for the stimuli with low (high) intensities. Thus, even if architectural constraints might prevent the AN2 neuron from achieving the theoretically optimal response curve, the selective increase (decrease) in mutual information provides a test for the infomax (selective coding) hypothesis.

### Adaptation to the Statistics of the Acoustic Environment

**Figure 9:**
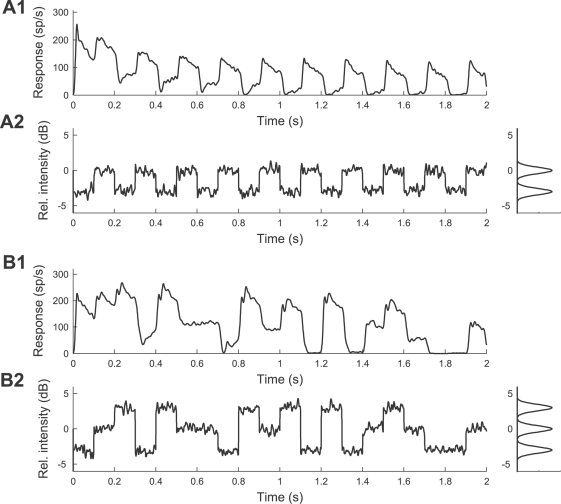
Representative responses of an AN2 cell (*T. leo*) to the amplitude-modulated noise stimuli of Figure 3C. (A1,A2) Bimodal stimulus distribution. The envelope of an amplitude-modulated stimulus and the distribution of the stimulus amplitude are shown in (A2), the corresponding instantaneous spike rate is shown in (A1). (B1,B2) Trimodal stimulus distribution. The envelope of an amplitude-modulated stimulus and the distribution of the stimulus amplitude are shown in (B2), the corresponding spike rate is shown in (B1). The stimuli were presented 45 times and the recorded spike trains (1 ms resolution) were convolved with a Gaussian kernel (σ = 5 ms). The instantaneous spike rates were estimated by averaging over the 45 repetitions.

Figure 9 shows example traces and the amplitude distribution of the bimodal and trimodal sound stimuli together with the corresponding neural responses of a typical AN2 cell (instantaneous firing rate). Adaptation leads to a decrease of the neural responses to 0 dB peak signals (drawn from the high amplitude and intermediate amplitude peak for the bimodal and trimodal distribution) with time.

We recorded responses from 25 AN2 cells for the two stimulus paradigms, 12 cells from *T. oceanicus* and 13 cells from *T. leo*. Since we found no significant differences in the adaptation and recovery time constants between the two species, we pooled the data from both species together for further analysis.

All response curves were quantified using sigmoid input-response functions (cf. Equation 5), and a Bayesian approach was used to determine the distribution of the corresponding parameters *A*, *B*_50_, and *C* (see Methods, Bayesian data analysis). Some cells did not show response saturation in the trimodal stimulus condition within the range of stimulus intensities we tested. In these cases, the uncertainty of the estimate of parameter *A* is high, and is reflected by a broad posterior distribution for this parameter. For most of the cells the test stimuli were strong enough to drive the cell to its maximum rate in both conditions. Although the response maximum occurs at higher stimulus intensities in the trimodal condition, we did not observe a systematic change of the saturation response. To fit the response curves, we assumed that the response maximum *A* has the same value for both stimulus conditions. Five cells were excluded from further analysis because the response curve corresponding to the expected parameter values (posterior means) did not provide a good fit to the data (the model accounted for less than 95% of the variability in the data; *R* ^2^<0.95). The further analysis is based on the remaining 20 cells.

**Figure 10:**
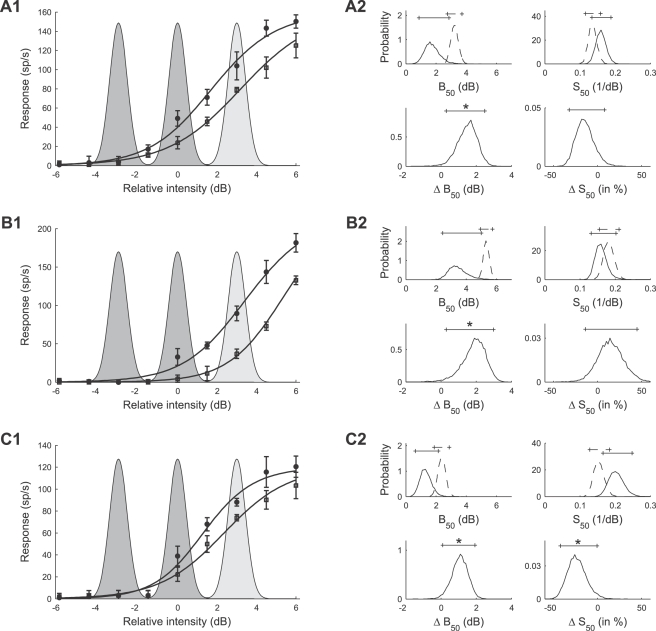
Typical examples of stimulus response curves after adaptation to the bimodal and to the trimodal stimulus distributions (A1,B1,C1) and posterior densities of the corresponding response curve parameters (A2,B2,C2). (A1,A2,C1,C2) Results for AN2 cells of *T. leo*. (B1,B2) Results for an AN2 cell of a *T. oceanicus*. (A1,B1,C1) Circles and squares denote the mean spike counts in a 200 ms time window of the test stimulus after adaptation to the bimodal and trimodal distributions, measured for 9 different relative intensities of the test stimulus (cf. protocol of Figure 3C). Error bars denote the standard deviation. Solid lines indicate the expected response curve, i.e., the response curve with the set of parameters with the mean value of the posterior distribution (see Methods, Bayesian data analysis). The shaded areas depict the intensity distribution of the stimuli (dark: bimodal stimulus distribution, light: additional peak of the trimodal stimulus distribution). (A2,B2,C2) Marginal posterior densities (cf. Methods, Bayesian data analysis) of the response curve parameters *B*_50_ (location) and *S*_50_ (slope). The posterior densities after adaptation to the bimodal (solid lines) and trimodal (dotted lines) stimulus distributions are shown in the top panels and the corresponding posterior densities of the changes (Δ*B*_50_, Δ*S*_50_) between stimulus conditions in the bottom panels. Solid (dotted) lines on top of the figures depict the 95% posterior intervals. Significant changes between stimulus conditions are indicated by a star.

A representative example of adapted response curves of an AN2 neuron is shown in Figure 10A1, where the input-response function is plotted for the parameters *A*, *B*_50_, and *C*, which correspond to the expected parameter values (posterior means). After adaptation to the bimodally distributed stimulus (filled symbols), the cell fired with 50% of its maximal rate (parameter *B*_50_) at about 1.75 dB. Adaptation to the trimodally distributed stimulus (open symbols) shifted the response curve to higher stimulus intensities while the slope of the response curve changed only slightly. In fact, the results of the Bayesian parameter estimation, depicted in Figure 10A2, revealed that the response curve parameter *B*_50_ significantly increased for the trimodal stimulus distribution. The mean of the posterior density changed from 1.74 dB to 3.23 dB (see Methods, Bayesian data analysis for the definition of statistical significance using Bayesian posterior intervals), while there was no significant change for the slope *S*_50_ (14% decrease from 0.160 dB^−1^). Figure 10B shows data from a second cell. The mean value of the parameter *B*_50_ is 3.47 dB for the bimodally adapted response curve, and increased by 1.86 dB through adaptation to the trimodally distributed stimulus. The increase of *B*_50_ was again significant. The slope increased by 15% (from 0.161 dB^−1^ for adaptation to the bimodal stimulus) but Bayesian analysis revealed that the increase in slope was not significant. Figure 10C shows data from a third cell. This cell showed a significant albeit less pronounced change in parameter *B*_50_ of +1.06 dB accompanied by a significant decrease in the slope *S*_50_ (decrease of the posterior mean by 23.5%).

#### Adaptation induced changes in the response curve parameters *B*_50_ and *S*_50_

**Figure 11:**
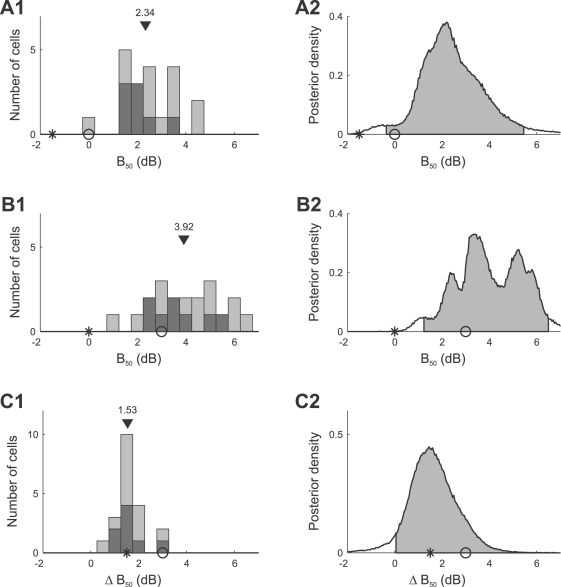
Summary of adaptation induced changes of the response curve parameter *B*_50_ for all 20 AN2 cells. Distribution of the mean values of the parameters *B*_50_ for individual cells (A1) and combined posterior density (see Methods, Bayesian data analysis) over all cells (A2) after adaptation to the bimodal stimulus distribution. (B1,B2) Distribution and combined posterior density of the parameter *B*_50_ after adaptation to the trimodal stimulus distribution. (C1,C2) Distribution and combined posterior density of the change of the parameter *B*_50_ between the two stimulus distributions. Symbols depict the values predicted by infomax (stars) and the selective coding hypothesis (circles). Triangles denote the median value. The distribution of cells that showed changes in *B*_50_ that were significant (Bayesian posterior intervals, see Methods, Bayesian data analysis) is marked black in (A1,B1,C1). Shaded areas depict the two-tailed 95% posterior intervals in (A2,B2) and the right-tailed 95% posterior interval in (C2).

Figure 11 summarizes the mean values of the posterior densities of the *B*_50_ parameters for all 20 AN2 cells. Figure 11A1 shows the values of parameter *B*_50_ after adaptation to the bimodal stimulus. The median value in the population is 2.34 dB (mean: 2.43 dB) and 2.02 dB (mean: 2.07 dB) for cells in which adaptation to the trimodal stimulus led to individual statistically significant changes in parameter *B*_50_ compared to adaptation to the bimodal stimulus (black distribution). The optimal *B*_50_ value predicted by the infomax principle is −1.5 dB (star), while selective coding predicts a *B*_50_ value of 0 dB (circle). The combined posterior distribution of *B*_50_ (cf. Methods, Bayesian data analysis) is shown in Figure 11A2 (mean: 2.43 dB). The measured *B*_50_ values are significantly larger than the values predicted by either hypotheses (infomax: −1.5 dB, selective coding: 0 dB). Figure 11B1 shows the histogram of *B*_50_ values after adaptation to the trimodal stimulus (median: 3.92 dB, mean: 4.04 dB; individually significant cells: median: 3.57 dB, mean: 3.69 dB). These values are significantly larger than the infomax prediction, but similar to the selective coding prediction (Figure 11B2). Figure 11C1 quantifies the difference of the parameter *B*_50_ between the two adaptation conditions. The median of the distribution of differences is 1.53 dB (mean: 1.61 dB). The right-tailed posterior interval in Figure 11C2 excludes the value 0 dB, indicating that adaptation to the trimodal stimulus significantly shifts the distribution of response curves towards higher signal intensities. Individual differences are statistically significant in 8 of 20 cells (see Methods, Bayesian data analysis); the median of the changes in these cells is 1.46 dB (mean: 1.62 dB). The observed shifts are smaller than expected for optimal selective coding (predicted shift: 3 dB), but compatible with the infomax principle (predicted shift: 1.5 dB). Due to the high absolute values of the thresholds, however, the response curves do not allow for reliable encoding of the whole stimulus range.

**Figure 12:**
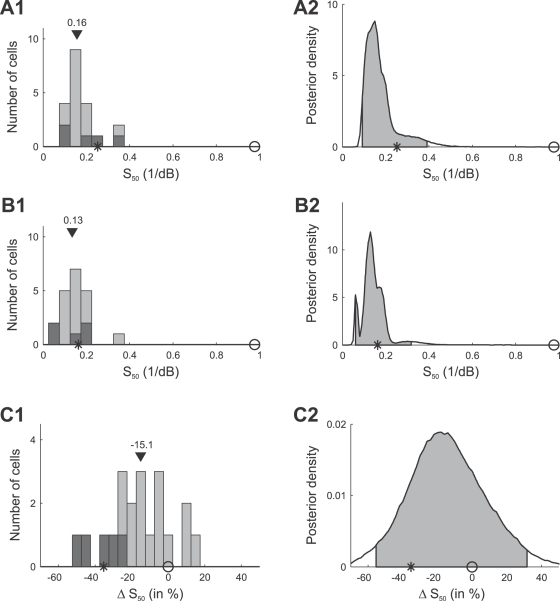
Summary of adaptation induced changes of the slope *S*_50_ of the response curves. Distribution of the mean values of the parameters *S*_50_ for individual cells (A1) and combined posterior density (cf. Methods, Bayesian data analysis) over all cells (A2) after adapting to the bimodal stimulus distribution. (B1,B2) Distribution and combined posterior density of the parameter *S*_50_ after adapting to the trimodal stimulus distribution. (C1,C2) Distribution and combined posterior density of the relative change of *S*_50_ between the two stimulus distributions. Symbols depict the values predicted by infomax (stars) and the selective coding hypothesis (circles). Triangles denote the median value. The distribution of cells that showed changes in *S_50_* that were significant (Bayesian posterior intervals, Methods, Bayesian data analysis) is marked black in (A1,B1,C1). Shaded areas in (A2,B2,C2) depict the 95% posterior intervals.

Figure 12 summarizes the mean estimates of the slope *S*_50_, for all 20 AN2 cells. The slopes in the bimodal adaptation paradigm (shown in Figure 12A1) have a median value of 0.16 dB^−1^ (mean: 0.17 dB^−1^), and are significantly smaller than the value of 0.98 dB^−1^ predicted by the selective coding hypothesis (Figure 12A2). The observed slopes *S*_50_ after adaptation to the trimodal stimulus are shown in Figure 12B, and the relative change of the slope compared to the bimodal paradigm is quantified in Figure 12C. The slope decreased for most cells (median: −15.1%, mean: −15.6%). Significant changes in *S*_50_ were found individually in 5 of 20 cells, and all of those cells showed decreases in slope. However, the changes are less pronounced than predicted by the infomax principle.

We conclude that the main difference between the response curves adapted to the bimodal vs. the trimodal stimulus distribution is the shift towards higher stimulus intensities and a reduction in slope. This shift, however, is less pronounced than predicted by optimal selective coding, and the observed decrease in slopes is smaller than predicted by the infomax principle and larger than expected by selective coding. Together with the fact, that the absolute thresholds are too high, these results seem not to favor either of the two coding hypotheses, if optimality is required.

#### Reliability of stimulus encoding

Adaptation in a biological system, which is constrained in multiple ways, may fall short of achieving the theoretical optimum, but may still lead to an improved representation according to the one or the other principle. In order to test for this, we calculate the mutual information between the stimulus and the neural response for the whole and for the high intensity part of the stimulus range. Therefore, 10000 samples were drawn from the joint posterior for the parameters *A*, *B*_50_, *C*, for each cell and for each stimulus condition, and the corresponding response curves were calculated using Equation 5. For each response curve, the joint distribution of stimulus and spike count was calculated assuming that spike counts are Poisson distributed with the underlying average spike count given by the response curve (cf. Methods, Numerical estimation of mutual information). Each of these joint distributions determines the mutual information (see Equations 13 and 14) that corresponds to a particular response curve.

We first consider the whole stimulus range from −4.5 dB to 4.5 dB and calculate the mutual information between the stimulus (trimodal distribution) and the neural response, for the response curves obtained after adaptation to the bimodal and trimodal stimulus distributions. According to the infomax principle the purpose of adaptation is to reliably encode the whole stimulus range and thus, the mutual information between the trimodal stimulus and the neural response should increase for the trimodally compared to the bimodally adapted response curve (predicted increase between 0.12 bit and 0.25 bit, depending on the maximum spike count; see Quantitative predictions).

**Figure 13:**
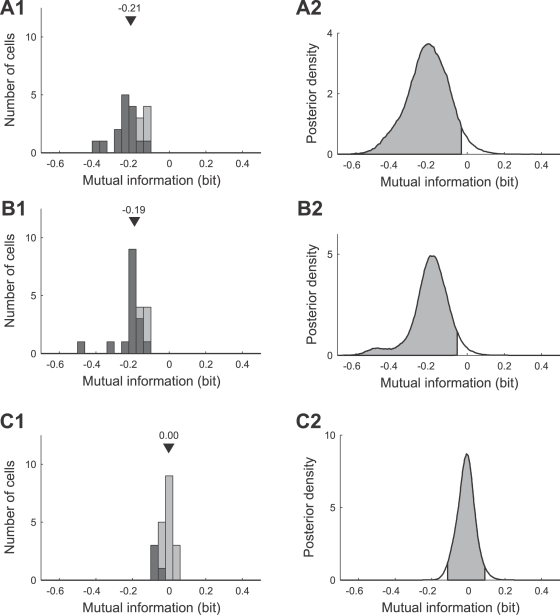
Adaptation induced changes in the mutual information between the stimulus and the neural response. (A1,A2) Distribution and combined posterior density of changes in the transmitted mutual information when considering the whole stimulus range (relative intensity from −4.5 dB to 4.5 dB) and the trimodal amplitude distribution. For each cell the change of the mutual information is calculated as the difference of the mutual information for the ‘trimodal’ (neural response adapted to the trimodal stimulus) and the ‘bimodal’ (neural response adapted to the bimodal stimulus) response curve. The distribution in (A1) is based on the mean values of changes in mutual information for individual cells. (B1,B2) Distribution and combined posterior density of changes in the transmitted mutual information when considering the stimulus range from −4.5 dB to 1.5 dB (including only the two low-intensity peaks of the trimodal stimulus distribution). (C1,C2) Distribution and combined posterior density of changes in the transmitted mutual information when considering the stimulus range from 1.5 dB to 4.5 dB (including only the high-intensity peak of the trimodal stimulus distribution). Triangles denote the median value. The distribution of cells that showed changes that were significant (Bayesian posterior intervals, Methods, Bayesian data analysis) is marked black in (A1,B1,C1). Shaded areas depict the left-tailed 95% posterior intervals in (A2,B2) and the two-tailed 95% posterior interval in (C2).

For the example neurons in Figure 10, however, we observed a significant decrease in mutual information, varying from a mean value of −0.183 bit (Figure 10A) to −0.372 bit (Figure 10B) and −0.187 bit (Figure 10C). This trend is confirmed by a full analysis of all 20 recorded AN2 cells (Figure 13A), which shows that mutual information decreased for all cells. The median is −0.21 bit (mean: −0.21 bit), and this decrease is significant (the left-tailed 95% posterior interval in Figure 13A2 excludes the value 0 dB). 15 of 20 cells showed an individually statistically significant decrease in mutual information (median −0.24 bit, mean −0.24 bit; black distribution in Figure 13A1). These findings provide strong evidence against the infomax principle.

In order to test the selective coding hypothesis, we calculated the mutual information separately for the stimulus range from 1.5 dB to 4.5 dB (high-intensity peak, ‘foreground’) and from −4.5 dB to 1.5 dB (low-intensity peaks, ‘background’; see Quantitative predictions) using Equation 14. For the cells shown in Figure 10, the mutual information decreased significantly by −0.184 bit (Figure 10A), −0.335 bit (Figure 10B) and −0.182 bit (Figure 10C) for the stimulus range from −4.5 dB to 1.5 dB. While the mutual information for the peak of the distribution with the highest intensity increased slightly by +0.018 bit for the cell shown in Figure 10A, in other cells, such as the ones shown in Figure 10B and 10C, the mutual information decreased not only for the ‘background’ but also for the loudest signal (−0.038 bit vs. −0.005 bit). However, these changes in encoding of the loudest signal were not statistically significant. Figure 13B summarizes the change in mutual information for the range from −4.5 dB to 1.5 dB for all 20 AN2 cells. Mutual information decreased significantly (the left-tailed 95% posterior interval in Figure 13B2 excludes the value 0 dB; median −0.19 bit, mean −0.20 bit), and the decrease was individually significant for 16 of the 20 cells. The information transmitted about the ‘loudest peak’ (Figure 13C), in the interval from 1.5 dB to 4.5 dB, remained constant (median 0.00 bit, mean −0.01 bit) and is not significantly different from zero (the 95% posterior interval in Figure 13C2 includes the value 0 dB). Although these results are consistent with the selective decrease of information for low-intensity stimuli, they contradict the selective coding hypothesis because information does not increase for high-intensity stimuli, as would be required for an improvement of the neural representation.

## Discussion

### Neurons in the Auditory Pathway of Crickets Adapt on Several Time Scales

In the cricket auditory system, time scales of adaptation observed at first level interneurons range from short (below 100 ms, AN1: [21]) over intermediate (ca. 300 ms; AN2: [22], receptors: [19]) to long time constants (ca. 10 s; AN2: [22], receptors: [19]). In the present study, we report firing rate adaptation with a time constant of about one second not reported before in the auditory ascending neuron AN2 of *T. oceanicus* and *T. leo* (Figure 6 and Table 1). At present the origin of adaptation in this small network is not known. There is likely a contribution to adaptation from the receptor neurons [19]. At the level of interneurons, both local cells (ON1, [11],[20]) as well as the ascending interneuron AN2 [22] exhibit long-lasting hyperpolarizations with intermediate time constants (approximately 5 s) that may reflect adaptation processes at the level of the spike-generator in these cells. The primary task of the first stage of auditory processing in crickets is to maintain and possibly to condense relevant information for object localization and recognition for higher computational centers in the brain. In this context, it is remarkable to note that already at the first synapse several time scales of adaptation can be observed, similar to those reported from vertebrate systems [36] as well as other sensory modalities [6]. The adaptation time scale we report here seems ideal for an adjustment of the coding scheme to the current sensory environment. However, we find no enhancement of information transfer in the neuron under study. We thus report the unusual case that adaptation seems to rather selectively suppress sensory coding instead of improving it.

### Bayesian Parameter Estimation

We characterized the neural response using a sigmoid response curve and estimated the model parameters from the measured spike counts using a Bayesian approach. An important feature of the analysis is that it yields the joint posterior distribution of the model parameters which allows us to calculate the posterior distribution and precise confidence limits of derived quantities such as mutual information (see [47] for the pitfalls of entropy estimation from undersampled discrete data such as ‘spike words’).

The Bayesian framework can also be applied to other experimental paradigms. One limitation of the approach presented here is that the calculation of the joint posterior density at a grid of points is only feasible for models with few parameters. However, for more complex models, approximations of the posterior densities can be obtained using Markov chain simulation [39].

### Adaptation and the Infomax Principle

We measured how adaptation changes the response curves of AN2 cells depending on different stimulus conditions (Figure 10) and found that the changes are not compatible with infomax (optimal coding of the entire stimulus range). In general, our results indicate a response threshold that is too high to allow for reliable encoding of the whole stimulus range (Figures 10 and 11). Most remarkably, adaptation reduced the amount of encoded information about the stimulus when considering the whole range of input signals (Figure 13A). This is in contrast to other studies (fly visual system [6],[23]; midbrain of guinea pigs [5]; inferior colliculus of cats [48]; songbird auditory forebrain [8]; rat barrel cortex [7]) that reported that stimulus encoding is compatible with infomax. However, the infomax principle, which considers sensory systems as communication channels that are optimized for preserving all information from the sensory input, may fail to explain neural coding when considering stages where actual processing of information takes place (instead of mere transmission). Indeed, a recent study by Ringach and Malone [49] has shown that neurons in the primary visual cortex of macaque maintain an operating point that does not maximize information transmission but is tuned to the detection of signals in background noise.

### Adaptation and the Selective Coding Hypothesis

We also tested if the response curve changes induced by adaptation are compatible with selective coding (reliable coding of the most intense signal while suppressing the ‘background’). Selective coding can be seen as the simplest form of separation of neural representations of discrete objects in multiple channels or ‘streams’ that has been found in higher auditory processing levels in vertebrates [35]. Neurons in the inferior colliculus of cats display the same firing pattern when a stimulus composed of a signal with or without background noise is presented, indicating a representation of the signal only [50], and in auditory cortex, neurons show locking to the amplitude modulations of a low level sound but not to the noise it is embedded in [34]. In insects, the principle has been found in bushcrickets, separating single males from background choruses [33] and has been suggested to be at work in crickets as well [20]. However, the selective coding principle is not clearly defined in an information theoretical framework. Here, we formalize the hypothesis and accordingly make two predictions about the change of information transfer with adaptation: (1) information conveyed about the ‘background’ should decrease, while (2) transmission for the high-intensity signals should increase. Indeed, we find that the neural output of the AN2 conveys less information about the first two peaks (‘background’) of the stimulus distribution (Figure 13B), supporting the selective coding hypothesis. However, if we take only the mutual information between the loudest part of the signal and the neural response into account, information rate remains nearly constant (Figure 13C). This contradicts the selective coding hypothesis.

### The Computational Role of Adaptation

Surprisingly, our results suggest that instead of improving sensory coding, adaptation in the AN2 decreases information transmission and leaves higher processing centers in the cricket brain with less (or at most equal) information, regardless of what part of the stimulus is considered. What could be the reason for this?

Adaptation leads to a selective decrease in mutual information for the low-intensity sounds, mainly by shifting the stimulus-response curves towards higher stimulus intensities. As a consequence, spike counts are reduced for low-intensity signals (cf. Figure 10). Through adaptation to the trimodal stimulus, average spike counts in response to the 0 dB test stimulus decreased for 18 of 20 cells (mean decrease: 42%, standard deviation: 31%) compared to the spike counts after adaptation to the bimodal stimulus. Thus, in an ecological setting, where background signals are present and foreground signals are changing their presence dynamically, background signals transmitted to downstream neurons by the AN2 will be reduced. We speculate that this might reduce the potential interference of ‘background’ and ‘foreground’ spikes in downstream processing. However, the observed response curves do not represent an optimal solution for the task of filtering out the most intense part of the stimulus.

Additionally, the algorithm behind adaptation could serve the goal of enhancing the representation of even louder signals, occurring with less probability. Examples, in which optimal coding is not used to maximize the average information gained about high probability stimuli include auditory receptors of locusts which seem to maximize the information gained about specific, but less often occurring aspects of the stimuli [51] and stimulus specific adaptation in single neurons of auditory cortex that leads to an enhanced representation of low-probability sounds deviating from the distribution of the surrounding signal [36]. This can be seen as a form of novelty detection, where part of the dynamic range is preserved for even louder sounds in a way that the sensory pathway is always able to detect brief, transient high-intensity signals [49]. In order to test if the representation of loud signals is enhanced, we calculated information transmission for a signal distribution that has an additional peak (modeled by a fourth Gaussian distribution with mean μ_4_ = +6 dB). Indeed, we find that adaptation increases mutual information in the stimulus range from 4.5 dB to 7.5 dB for all cells. This increase is significant in 6 of 20 cells, but it is not statistically significant on the population level (the 95% interval of the combined posterior density ranges from −0.015 bit to 0.243 bit).

In this context, it should also be noted that the AN2 neuron in crickets may serve several functions. Under most stimulus conditions, relatively low firing rates will likely monitor slowly changing signals as observed in the present study (up to about 5 Hz). The AN2, however, can also operate in a burst mode with high intra-burst firing rates for the detection of bat calls [16] for which our analysis is not appropriate. Nevertheless, low firing rates are likely to transmit relevant information since input-response curves built from spike counts similar to those in the present study are maintained at somewhat higher thresholds in wingless cricket morphs that are not at risk from bat predation [18]. In addition to its relevance for slow signal features, the adaptation time course reported here is likely to adjust the operating point of the faster response dynamics (i.e., bursts). Apart from possible physiological limitation, the findings we report here could be the result of a trade-off between setting the operating regime for the bursting mode on the one hand and suppression of background noise on slower time scales on the other hand.

Generally, a neural system may achieve improved performance by means of different mechanisms as has been shown in a recent modeling study [52]. Depending on the specific physiological constraints, the resulting neural representation can be optimal or a trade-off between optimality and the flexibility of the neural circuit. Indeed, we found that, for example, the slope of the stimulus-response curve is not steep enough for optimal encoding of only the loudest peak of the bimodal or trimodal stimulus distribution. Possibly the neural gain can only increase to a limited value, leading to a decrease—rather than an increase—in information transmission for the chosen experimental paradigm.

Although we cannot give a conclusive answer on what the adaptation-induced selective suppression in the AN2 serves for yet, the paradigm we propose here is rather general and should be applicable to other sensory systems as well. Importantly, our paradigm allows quantifying the improvement in neural coding without requiring that neural response curves achieve optimality. Measuring the information between the proposed relevant stimulus and the neural output allows testing for different hypotheses on what a sensory pathway actually adapts to. Ultimately, testing various hypotheses on different stimulus ensembles will yield important insights on what is or what is not the relevant part of a sensory environment for a given sensory unit.
